# Terahertz Dispersion Characteristics of Super-aligned Multi-walled Carbon Nanotubes and Enhanced Transmission through Subwavelength Apertures

**DOI:** 10.1038/s41598-018-20118-5

**Published:** 2018-02-01

**Authors:** Yue Wang, Guangwu Duan, Liying Zhang, Lihua Ma, Xiaoguang Zhao, Xin Zhang

**Affiliations:** 1 School of Science, Xi’an University of Technology, Xi’an, 713300 China; 2 Department of Mechanical Engineering, Boston University, Boston, MA 02215 USA; 3 Key Laboratory of Engineering Dielectrics and Its Application, Ministry of Education, Harbin University of Science and Technology, Harbin, 150080 China

## Abstract

The terahertz (THz) dielectric properties of super-aligned multi-walled carbon nanotube (MWCNT) films were characterized in the frequency range from 0.1 to 2.5 THz with terahertz time-domain spectroscopy. The refractive index, effective permittivity, and conductivity were retrieved from the measured transmission spectra with THz incident wave polarized parallel and perpendicular to the orientation of carbon nanotubes (CNTs), and a high degree of polarization dependence was observed. The Drude-Lorentz model combined with Maxwell-Garnett effective medium theory was employed to explain the experimental results, revealing an obvious metallic behavior of the MWCNT films. Moreover, rectangular aperture arrays were patterned on the super-aligned MWCNT films with laser-machining techniques, and the transmission measurement demonstrated an extraordinarily enhanced transmission characteristic of the samples with incident wave polarized parallel to the orientation of the CNTs. Surface plasmon polaritons were employed to explain the extraordinarily enhanced transmission with high accuracy, and multi-order Fano profile was applied to model the transmission spectra. A high degree of agreement was exhibited among the experimental, numerical, and theoretical results.

## Introduction

The discovery of extraordinarily enhanced transmission of incident electromagnetic radiation through periodic arrays of subwavelength patterns perforated on metallic films by Ebbesen and co-workers has inspired tremendous interest in exploring the underlying physics and potential applications^1–5^. Various experimental and theoretical studies have been conducted to explain the mechanism of the enhanced transmission phenomenon, and it is widely believed that this enhanced transmission is due to the resonant excitation of surface plasmon polaritons (SPPs) and waveguide mode resonance^6–9^. Moreover, various geometries of periodic arrays of subwavelength apertures have been extensively studied for this enhanced transmission^10–14^. In this paper, we focused on the experimental and theoretical study of the enhancement of radiation transmission through two-dimensional asymmetric periodic rectangular aperture arrays in the terahertz (THz) regime.

Besides metals, many other materials such as semiconductors, transparent conducting oxides, and novel carbon-based materials can be  consdiered as conductive media to support the formation of THz SPPs^15–22^. These emerging materials enable new possibilities to inspire fundamental research and potential applications in the THz regime. Recently, there has been an increasing interest in exploring the optical and electrical properties of aligned and non-aligned carbon nanotube (CNT) films in the THz regime^23,24^. Specifically, compared with non-aligned CNTs, super-aligned CNTs exhibit strong THz polarization anisotropy among other characteristic properties^25,26^.

In this paper, we investigated the anisotropic electric and optical properties of super-aligned multi-walled carbon nanotube (MWCNT) films on silicon substrates using the THz time-domain spectroscopy (THz-TDS). The transmission field strength was measured with incident THz wave polarized parallel (*E*_**//**_) and perpendicular (*E*_**⊥**_) to the orientation of the CNTs. In the entire frequency range of our experiment (0.1–2.5 THz), the measured data agreed well with the theoretical results based on the Maxwell-Garnett (M-G) effective medium theory combined Drude-Lorentz (DL) model. In addition, the experimentally extracted refractive index and effective permittivity reveal that our MWCNT films possess a prominent anisotropy and metallic behavior. After studying the optical properties of the MWCNT films, we present experimental and simulation results of the polarization-dependent SPP-enhanced transmission of THz pulses through asymmetric rectangular aperture arrays patterned on MWCNT films with two periodicities in the frequency from 0.1 to 2.0 THz. Simulation results of the enhanced transmission spectra matched well with the experimental results. While keeping the long axis of the aperture perpendicular to the orientation of CNTs, we observed a significant peak in the transmission magnitude in the case of THz pulses polarized parallel to the orientation of the CNTs. This result demonstrates that the extraordinary enhanced transmission depends on the polarization direction of the incident THz wave.

## Experiments

**Figure 1:**
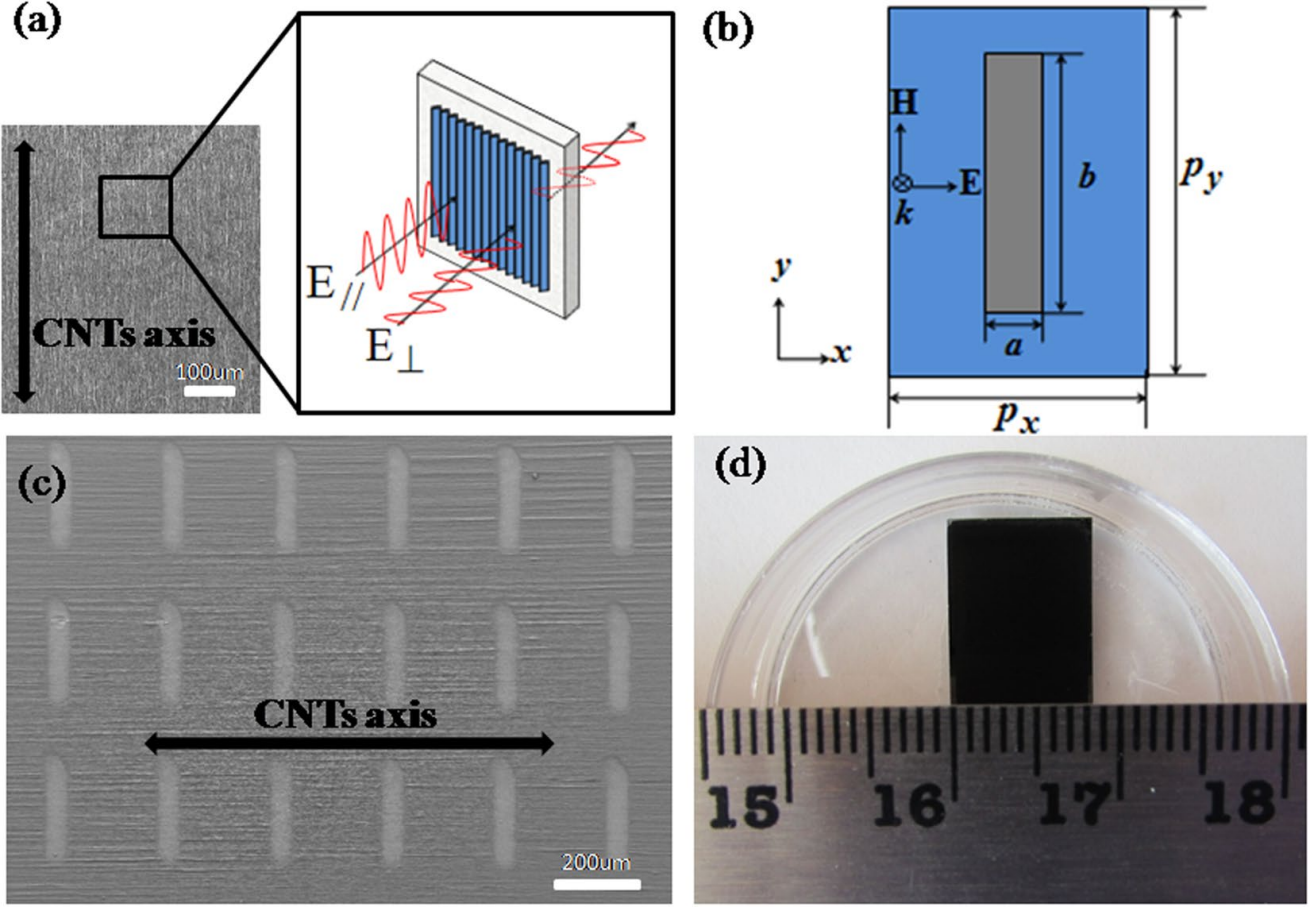
(**a**) SEM image of aligned multi-walled carbon nanotubes (MWCNTs); inset is the illustration of the nanotube alignment direction and the THz polarization direction. (**b**) Schematic drawing of a rectangular aperture unit. (**c**) SEM image of a 3-µm-thick MWCNT film with periodic rectangular aperture arrays with long axis perpendicular to the orientation of the CNTs. (**d**) Optical image of a fabricated sample.

In this study, the super-aligned MWCNT films, synthesized via low pressure chemical vapor deposition^27,28^, were directly drawn out from the sidewall of super-aligned CNT arrays on a 750-µm-thick silicon substrate. Figure 1(a) shows a scanning electron microscope (SEM) image of the MWCNTs indicating the high degree of parallel alignment of the CNTs in the film. The inset is the illustration of the incident THz pulses polarized parallel (*E*_**//**_) or perpendicular (*E*_**⊥**_) to the orientation of the nanotubes. The two-dimensional asymmetric periodic rectangular aperture array structures utilized in this experiment were patterned on 3-µm-thick MWCNT films with a laser-machining technique. As depicted in Fig. 1(b), the width and length of the single rectangular aperture is *a* and *b*, respectively, and the periodicities are *P*_*x*_ and *P*_*y*_ along the *x*(short) and *y*(long) axis, respectively. In our experiment, while keeping *a* = 50 µm and *b* = *P*_*x*_, we modified the periodicity between apertures: *P*_*x*_ = 150 µm, *P*_*y*_ = 225 µm and *P*_*x*_ = 250 µm, *P*_*y*_ = 375 µm. Figure 1(c) shows the top-down SEM image of a MWCNT film with periodic rectangular aperture arrays (*P*_*x*_ = 250 µm) indicating that the long axis of the aperture is perpendicular to the orientation of the CNTs, and Fig. 1(d) shows the image of a fabricated sample. A standard THz-TDS system (CIP-TDS, Daheng New Epoch Technology Inc.) was employed to investigate the THz transmission properties of these MWCNT samples. The entire system was sealed in a dry nitrogen gas environment to reduce the signal attenuation caused by moisture.

## Results and Discussions

**Figure 2:**
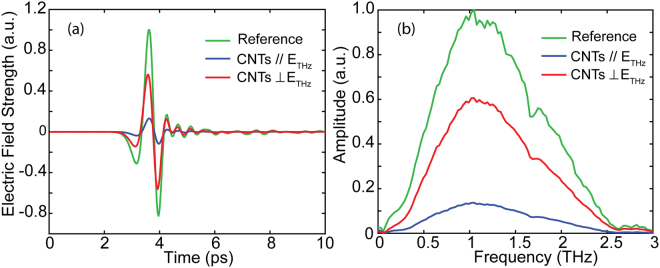
(**a**) Transmitted THz pulses in the time domain from the reference silicon substrate (green curve) and from the MWCNT films with the incident THz pulses polarized parallel (blue curve) and perpendicular (red curve) to the orientation of the CNTs. (**b**) The corresponding amplitude spectra in frequency domain.

It is critical to investigate the dielectric properties of the unpatterned MWCNT films before studying the enhanced transmission properties of the patterned MWCNT films. Figure 2(a) plots the reference signal measured with a bare silicon substrate and the transmission signals with incident THz pulses polarized parallel and perpendicular to the orientation of the CNTs. Subsequently, the time-domain transmission signals were fast Fourier transformed to the frequency domain and they are depicted in Fig. 2(b). Figure 2 shows the high anisotropy in THz transmission responses of MWCNT films. The anisotropic properties of the super-aligned MWCNT films arise from the unique structure with ultra-high aspect ratio and the super-aligned orientation of the CNTs. The CNTs in the sample are featured with a diameter in nanometer scale and length in centimeter scale, enabling its anisotropic electric properties in microscale, which demonstratesd a high conductivity along the longitudinal direction, while a low conductivity along the transversal direction. Furthermore, the carbon nanotubes were fabricated with a super-aligned orientation resulting in the anisotropic properties in the macroscale exhibiting a 6:1 contrast ratio in the transmission amplitude of THz pulses with two orthogonal polarizations, as shown in Fig. 2(b). Multiple transmission measurements were performed and the spectra was highly repeatable. Subsequently, the frequency-domain signals were normalized to the reference signal as shown in^29–31^:

1 $$\frac{{\tilde{E}}_{s}(\omega )}{{\tilde{E}}_{r}(\omega )}=\rho (\omega )\cdot {e}^{-j\phi (\omega )}$$

in which ${\tilde{E}}_{s}(\omega )$ and ${\tilde{E}}_{r}(\omega )$ are the complex frequency spectra of the sample and the reference respectively, *ρ*(*ω*) is the ratio of magnitude, and *φ*(*ω*) is the phase difference. Using the relationship of $\rho (\omega )=\exp (-\frac{k}{c}\omega d)$ and $\phi (\omega )=\frac{2\pi }{\lambda }nd$, the real part (*n*) and imaginary part (*k*) of the complex refraction index can be acquired.

**Figure 3:**
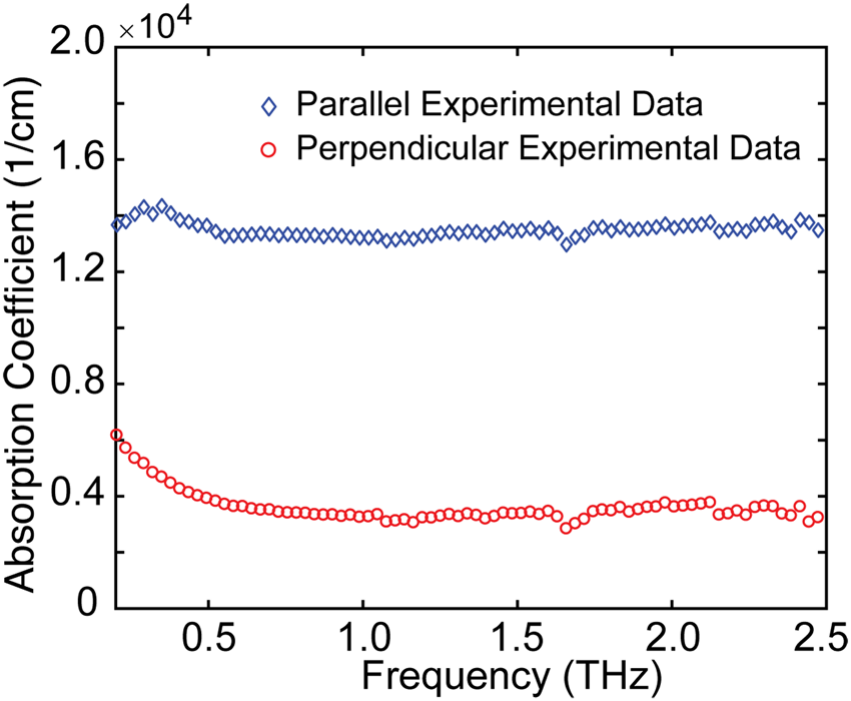
Power absorption coefficient spectra for MWCNT films. Diamonds represent the experimental data in the parallel case, while circles represent experimental data in the perpendicular case.

In Fig. 2, the measured transmission amplitude is related to the power absorption coefficient $\alpha (\omega )$, which depends on the imaginary part of the complex refractive index (*k*) given by $\alpha (\omega )=2\omega k/c$. The diamonds and circles in Fig. 3 show the measured power absorption coefficient of the MWCNT films. As can be observed in Fig. 3, when the frequency increases from 0.5 to 2.5 THz, the power absorption coefficients in *E*_**//**_ and *E*_**⊥**_ directions are both nearly constant. In addition, the power absorption coefficient in *E*_**//**_ direction is much larger than that in *E*_**⊥**_ direction. The values approximate 1.35 × 10^4^/cm and 3 × 10^3^/cm in *E*_**//**_ and *E*_**⊥**_ directions, respectively, which also demonstrate extremely high anisotropy.

**Figure 4:**
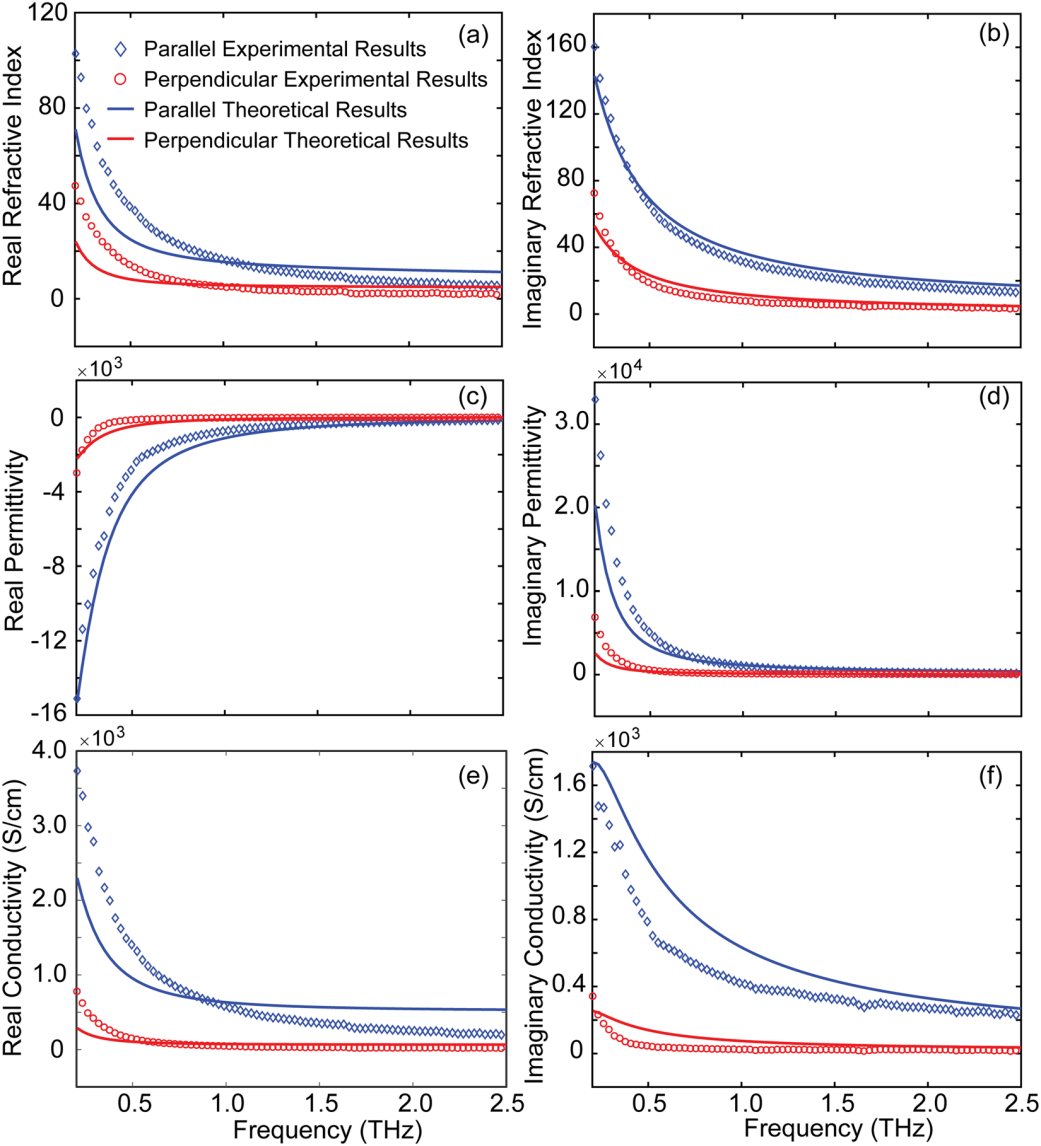
Comparison of measurement results (diamonds and circles) with theoretical results (solid lines), (**a**) Real part of the refractive index, (**b**) imaginary part of the refractive index, (**c**) real part of the effective permittivity (**d**) imaginary part of the effective permittivity, (**e**) real part of the conductivity, and (**f**) imaginary part of the conductivity. Diamonds and blue lines indicate the parallel direction, while circles and red lines indicate the perpendicular direction.

In Fig. 4(a) and (b), the diamonds and circles represent the retrieved real and imaginary refractive indices as a function of frequency from the measurement results. It indicates that the refractive indices decrease with increasing frequency and saturate at high frequencies for both *E*_**//**_ and *E*_**⊥**_. The refractive indices approached 5.3 and 2.2 respectively, corresponding to a real part effective dielectric constant of 5.3^2^ and 2.2^2^ in the two orientations. Furthermore, the dielectric function for MWCNT films is related to the complex refractive index  with the following relationship:

2 $$\varepsilon ={\varepsilon }_{\infty }+i\sigma /\omega {\varepsilon }_{0}={(n+ik)}^{2}$$

where *ε*_∞_ is the dielectric constant of CNT films at infinitely large frequencies, and *ε*_0_ is the free-space permittivity. The retrieved permittivity is plotted in Fig. 4(c) and (d).

The real and imaginary parts of the conductivity can be acquired from $${\sigma }_{real}=2\omega nk{\varepsilon }_{0}$$ and $${\sigma }_{imag}=\omega {\varepsilon }_{0}$$$$[{\varepsilon }_{\infty }-({n}^{2}-{k}^{2})]$$, as shown in Fig. 4(e) and (f). Importantly, Fig. 4(c) reveals that the real part of the effective permittivity $${\varepsilon }_{real}={n}^{2}-{k}^{2}$$ in the two cases is negative over a wide frequency range from 0.2 to 2.5 THz, which is a critical condition for a medium to support propagating SPPs. In order to understand the effective complex dielectric constant, the M-G model was introduced^32^:

3 $${\varepsilon }_{eff}={\varepsilon }_{i}\frac{(1-N)(1-f){\varepsilon }_{i}+[N+f(1-N)]{\varepsilon }_{m}}{(fN+1-N){\varepsilon }_{i}+N(1-f){\varepsilon }_{m}}$$

where *ε*_*i*_ is the background permittivity constant, *ε*_*m*_ is the effective permittivity constant of MWCNT films, and *f* and *N* are the filling factor and geometrical factor of the M-G model, respectively. In this study, we assume that the background is filled with air. Therefore, using M-G effective medium model to explain this composite system is necessary. When the orientation of the MWCNTs to the THz polarization changes, the geometrical factor ${N}$ (Table 1) has different values.

**Table 1:** Best-fit parameters used in combined M-G and DL model for the theoretical curves presented in Fig. 4(a–f).

Parameter	*ω/*2π (THz)	γ/2π (THz)	*ω*_pj_*/*2π (THz)	*ω*_j_*/*2π (THz)	γ_j_/2π (THz)	*f*	*N*	*ε*_∞_
Parallel	39.30	0.18	785.75	6.21	532.90	0.80	9.99 × 10^−6^	28.09
Perpendicular	13.13	0.13	463.97	8.91	1477.50	0.80	1.99 × 10^−4^	4.84

To better evaluate the effective dielectric constant of MWCNTs, i.e., *ε*_*m*_, we used a combined Drude and Lorentz oscillation model:

4 $$\,{\varepsilon }_{m}={\varepsilon }_{\infty }-\frac{{\omega }_{p}^{2}}{\omega (\omega +i\gamma )}+\sum _{j}\frac{{\omega }_{pj}^{2}}{({\omega }_{j}^{2}-{\omega }^{2})-i{\gamma }_{j}\omega }$$

where *ε*_∞_ is the dielectric constant of MWCNT films at infinity, *γ* is the damping rate, *ω*_*p*_ and *ω*_*j*_ represent the plasma and phonon frequency, respectively, and *ω*_*pj*_ and *γ*_*j*_ represent the center frequency and damping frequency of the Lorentz oscillator, respectively. The theoretical curves were fitted to the measurement data with parameters listed in Table 1.

The theoretical effective permittivity and conductivity of the MWCNT films from Eqs (2–4) with parameters in Table 1 are plotted in Fig. 4(c–f) with the blue lines and red lines for the case of *E*_**//**_ and *E*_**⊥**_. As shown in Fig. 4(e) and (f), similar to the refractive indices, the conductivity decreases with increasing frequencies in both *E*_**//**_ and *E*_**⊥**_ directions.  Importantly, the conductivity in *E*_**//**_ direction is much larger than that in *E*_**⊥**_ direction, demonstrating a prominent anisotropy and metallic behavior in our MWCNT films. In addition, the anisotropic attribute of the MWCNT was also supported by the extracted material properties in Fig. 4(a–d). The value of plasma frequency of the MWCNT films is $\frac{{\omega }_{p}}{2\pi }=13.1$ THz and 39.3 THz in *E*_**⊥**_ and *E*_**//**_ directions, respectively, falling between doped semiconductors and perfect metals. Furthermore, in terms of the carrier density, the higher plasma frequency in *E*_**//**_ direction indicates a larger free electron density and conductivity along the CNTs. Due to the atomic arrangement with high aspect ratio, the electromagnetic properties of the MWCNT films are not only depended on the atomic arrangement but also on the orientation of the CNTs. The orientation of carbon nanotubes presented in both directions in the MWCNT films (even very minors along one direction, it still cannot be eliminated), and the MWCNT films were filled with air. It was difficult to measure the apparent density of the film. Therefore, the samples reported in this work have to be treated as composite material, which caused the difference between the experimental results and theoretical results. The difference can be decreased by improving the fabrication process to eliminate the carbon nanotube orientated along the minor direction.

**Figure 5:**
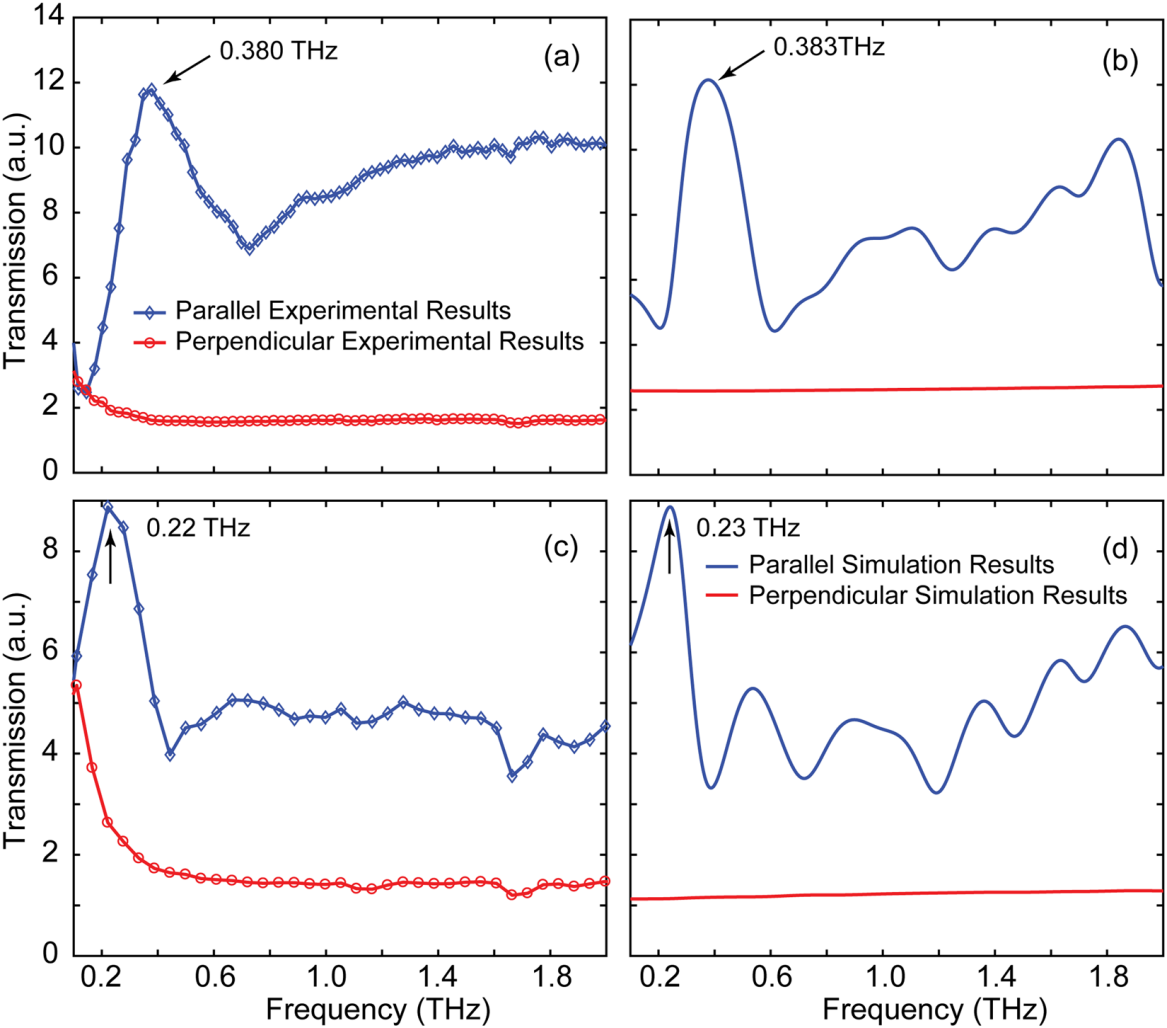
Experimental (**a** and **c**) and simulated (**b** and **d**) transmission spectra for two different periodicities along *x*-direction for the 3-µm-thick MWCNT film: *P*_*x*_ = 150 µm in (**a**) and (**b**), *P*_*x*_ = 250 µm in (**c**) and (**d**). Diamonds and blue lines represent the transmission for polarizations parallel to the short axis of the rectangular apertures, while circles and red lines represent the transmission for perpendicular polarizations.

We fabricated rectangular aperture arrays with two different periodicities (*p*_x_ = 150 μm and *p*_y_ = 250 μm; *p*_x_ = 250 μm and *p*_y_ = 375 μm) on the MWCNT films, as schematically shown in Fig. 1(b). Numerical simulations of the transmissions spectra of those samples were performed using the commercial software CST Microwave Studio with modeled permittivity in Fig. 4(c) and (d). A plane wave THz pulse was normally incident on a unit cell of a rectangular aperture to simulate the transmission spectrum. Periodic boundary conditions were applied along *x* and *y* axes perpendicular to the pulse propagation direction. The transmission signal was fast Fourier transformed to the frequency domain and normalized to the reference signal, which was obtained as the transmission through unpatterned films. The results are shown in Fig. 5.

In Fig. 5, the simulated transmission characteristics demonstrate a similar trend with the experimental results at the resonant frequencies. We note that there are differences between the experimental and simulated results at high frequencies. We consider the disagreement is due to the bulk approximation of the dielectric properties of the MWCNT films in simulations. In the simulation model, the CNT film has been considered as macroscopic bulk material rather than nanotubes with specific shapes and orientations. In reality, however, the CNT shape and orientation produce clear anisotropic conductivities. Furthermore, the unit cell in the simulation limits the accessible spatial extent of impedance-matched electrical pathways, which are thought to be responsible for the multiple-oscillation of the transmission spectra at higher frequencies. At the perpendicular polarization, the transmission remains almost unchanged at frequencies over 0.2 THz in both simulation and experimental results. However, we note that the transmitted signal in experimental results is slightly stronger than that of the simulation results at frequencies lower than 0.2 THz. This discrepancy could be attributed to the superposition effect of noise and the weak THz transmission energy at the low-frequency range shown in Fig. 2(b). It is worth noting that, the spectral transmissions are clearly affected by the incident THz polarization. In *E*_**//**_ polarization, the samples exhibit an obvious transmission enhancement phenomenon and the resonance peaks occur at 0.38 THz and 0.22 THz, while the transmission is almost completely suppressed in *E*_**⊥**_ polarization. Importantly, the amplitude enhancement ratio between patterned and unpatterned MWCNT films at 0.38 THz and 0.22 THz can reach 12 and 9, respectively. In addition, the transmission minima appear at 0.72 THz and 0.44 THz in the spectra due to Wood’s anomaly for the case of *E*_**//**_^33,34^. The extraordinarily enhanced transmission can be explained in terms of SPPs excited at the interfaces of the rectangular aperture arrays and the silicon substrate. For normal incidence, the dispersion relation is approximately given by^2^:

5 $$\,{k}_{sp}={k}_{0}\sqrt{\frac{{\varepsilon }_{1}{\varepsilon }_{eff}}{{\varepsilon }_{1}+{\varepsilon }_{eff}}}$$

where *k*_0_ = 2*πf/c* is the free-space wave vector, *ε*_1_ and *ε*_eff_ are the permittivity of dielectric medium and MWCNT films, respectively. For the 2D rectangular aperture array with periodicity along *x*-axis (*P*_*x*_), the SPPs wave vector is calculated as^1^:

6 $$\,{{\boldsymbol{k}}}_{sp}={{\boldsymbol{k}}}_{0}sin\theta \pm m{{\boldsymbol{G}}}_{x}\pm n{{\boldsymbol{G}}}_{y}$$

where *m* and *n* are integers, ***G***_*x*_ and ***G***_*y*_ are the reciprocal lattice vectors with |***G***_*x*_| = 2*π/p*_*x*_ and |***G***_*y*_| = 2*π/p*_*y*_. When THz wave is incident normally, $sin\theta =0$. By solving Eqs (5) and (6), the complex resonance frequency based on SPPs modes is given by:

7 $$f=\frac{c}{{p}_{x}}\sqrt{{m}^{2}+\frac{4}{9}{n}^{2}}\sqrt{\frac{{\varepsilon }_{1}+{\varepsilon }_{eff}}{{\varepsilon }_{1}{\varepsilon }_{eff}}}$$

The real part of *f* corresponds to the resonance frequency, and the imaginary part of *f* is related to the internal damping corresponding to the coupling between the CNT’s absorption loss and the surface plasmon. The calculated resonant frequencies of the samples with two different periodicities along *x*-axis (*P*_*x*_ = 150 µm, 250 µm) based on SPPs for the MWCNTs/Si interface are 0.38 THz [0, ±1] and 0.22 THz [0, ±1], respectively, which agrees well with the experimental results.

**Figure 6:**
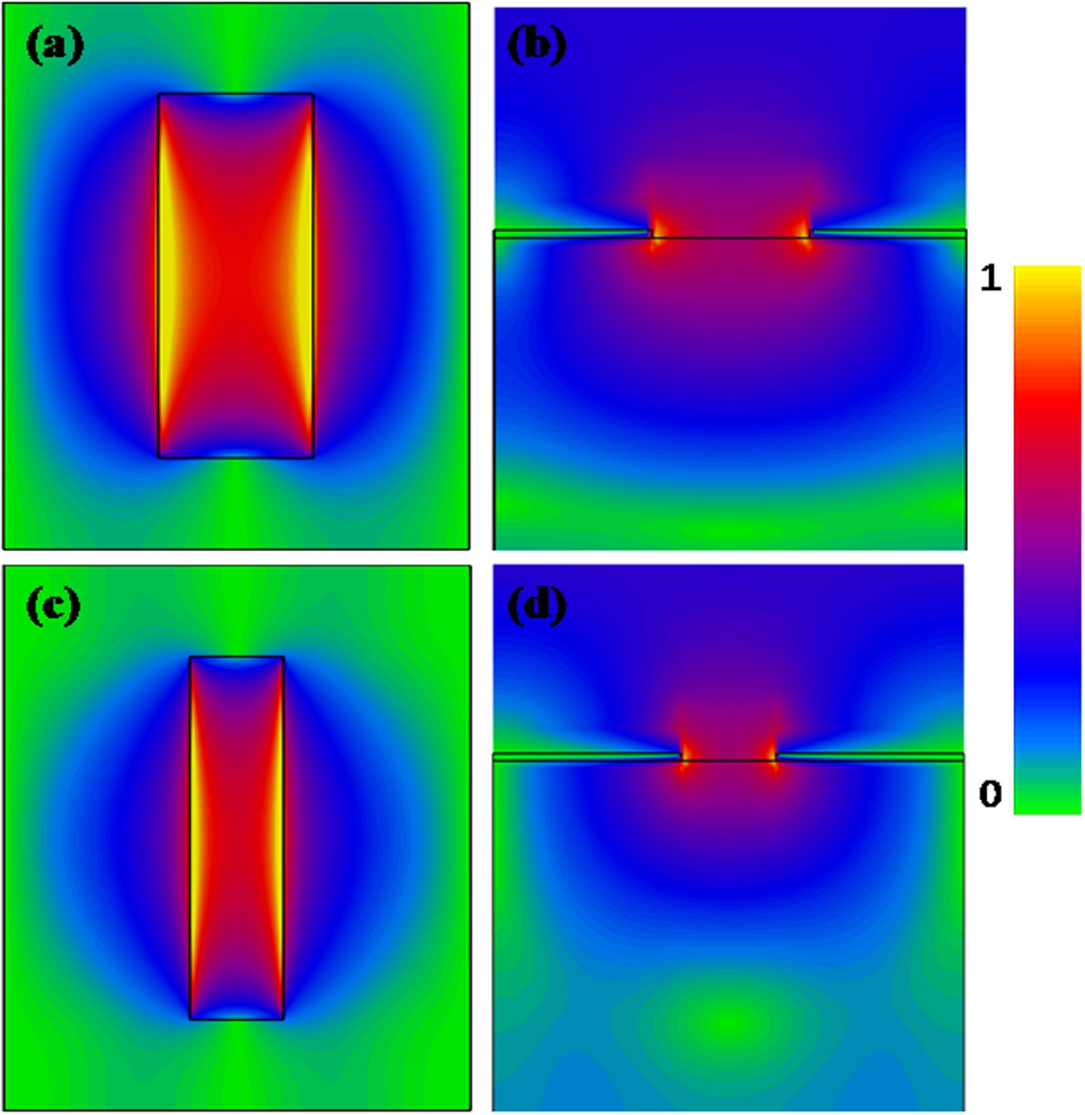
Simulated electric field distribution in the *xy* plane through the rectangular aperture for periodicities along *x*-axis of (**a**) 150 µm, and (**c**) 250 µm at peak frequencies. (**b**) and (**d**) are the simulated electric field distribution in the *yz* plane corresponding to (**a**) and (**c**).

To further understand the enhanced resonance transmission, we simulated the electric field distribution of each resonant mode. The electric field distributions at 0.38 THz and 0.22 THz of those asymmetric rectangular apertures with periodicities of 150 µm and 250 µm along short axis are shown in Fig. 6. In the case of the rectangular aperture, a distinct electric field enhancement occurs inside the rectangle at the resonant frequencies, which reflects the fact that the enhancement transmission arose from the resonance of SPPs.

From Fig. 5, it can be seen that the resonance exhibits a distinctly asymmetric line shape. This phenomenon is related to the interfering in the transmission process and agrees well with the Fano profile^35,36^. Fano resonance has been found in a number of metallic films, photonic crystals, plasmonics nano structures^37,38^, and so on, but seldom in CNT films. Herein, we prove that Fano resonance can also provide useful insight in treating the transmission of THz pulses through patterned MWCNT films. Such Fano-like resonance can be attributed to the interference between non-resonant and resonant scattering in the periodic aperture arrays. The classical Fano model is defined by^11,35^:

8 $$T(\omega )={t}_{0}+{t}_{i}\frac{{[\frac{2(\omega -{\omega }_{0})}{\gamma }+f]}^{2}}{{\frac{4(\omega -{\omega }_{0})}{{\gamma }^{2}}}^{2}+1}$$

where *ω*_0_, *ω*, and *γ* are the resonance frequency, signal frequency and the spectra linewidth, respectively. *f* is the asymmetric parameter that defines the degree of asymmetry, indicating a ratio of the transition probabilities to the discrete state and to the continuum state. *t*_0_ and *t*_i_ are slowly varying transmission and background transmission, respectively. In our case, considering the resonant state associated with surface plasmons from the CNT films, the entire transmission of the periodic aperture arrays can be expressed by the modified multi-order Fano profile^39^, given by:

9 $$\begin{array}{c}T({\rm{\omega }})={t}_{0}+{a}_{1}\{\frac{{[\frac{2(\omega -{\omega }_{1})}{{\gamma }_{1}}+{f}_{1}]}^{2}}{{\frac{4(\omega -{\omega }_{1})}{{\gamma }_{1}^{2}}}^{2}+1}-1\}+{a}_{2}\{\frac{{[\frac{2(\omega -{\omega }_{2})}{{\gamma }_{2}}+{f}_{2}]}^{2}}{{\frac{4(\omega -{\omega }_{2})}{{\gamma }_{2}^{2}}}^{2}+1}-1\}\\ \,\,\,\,\,\,\,\,\,\,\,\,\,+{a}_{3}\{\frac{{[\frac{2(\omega -{\omega }_{3})}{{\gamma }_{3}}+{f}_{3}]}^{2}}{{\frac{4(\omega -{\omega }_{1})}{{\gamma }_{3}^{2}}}^{2}+1}-1\}\end{array}$$

where *a*_i_ (*i* = 1, 2, and 3) is a weighting factor for each resonant mode. It also modifies the intensity of the transmission.

**Figure 7:**
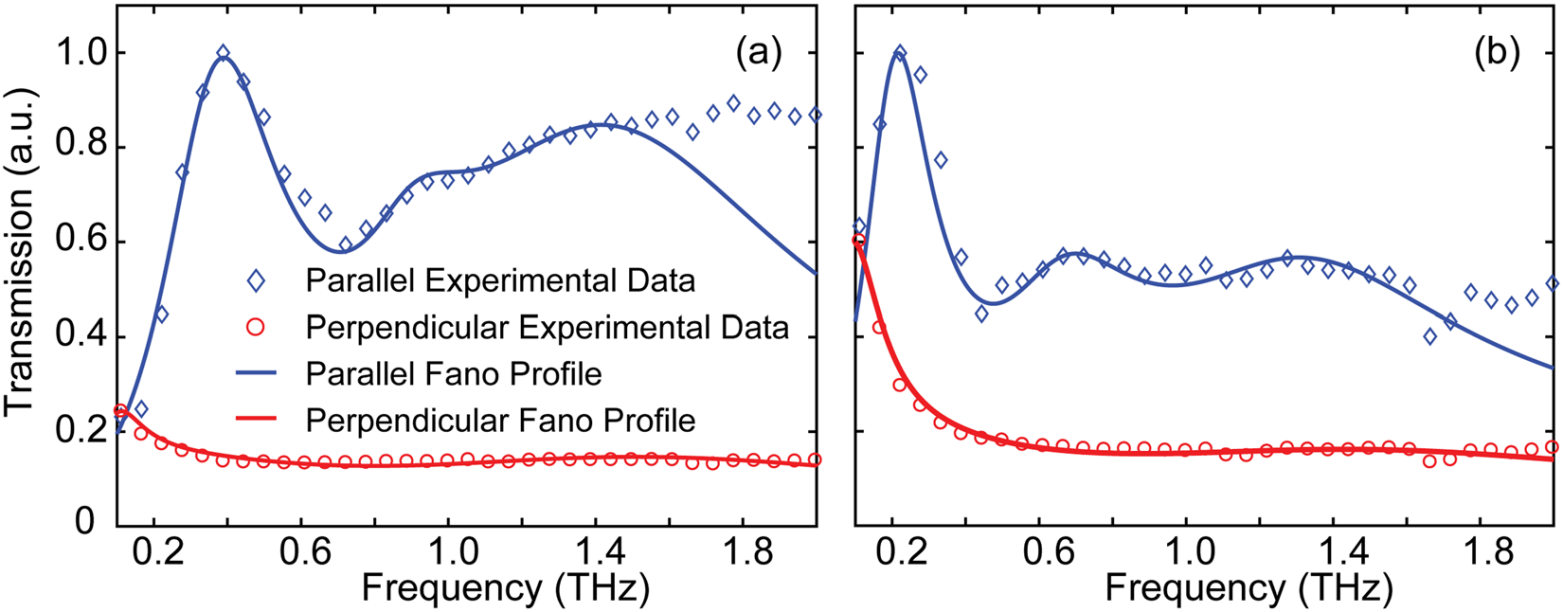
Normalized transmission spectra fitted by Fano model with various parameters. (**a**) Sample with *p*_x_ = 150 μm. The Fano model parameters: *t*_0_ = 0.01, *ω*_1_/2π = 0.351 THz, *ω*_2_/2π = 0.885 THz, and *ω*_3_/2π = 1.356 THz; *f*_1_ = 5.78, *f*_2_ = 4.24, and *f*_3_ = 5.19; γ_1_/2π = 0.385 THz, γ_2_/2π = 0.37 THz, and γ_3_/2π = 1.19 THz; *a*_1_ = 2.76×10^−2^, *a*_2_ = 1.22×10^−2^, and *a*_3_ = 2.62×10^−2^. (**b**) Sample with *p*_x_ = 250 μm. The Fano model parameters: *t*_0_ = 0. 1, *ω*_1_/2π = 0.196 THz, *ω*_2_/2π = 0.645 THz, and *ω*_3_/2π = 1.256 THz; *f*_1_ = 5.77, *f*_2_ = 4.36, and *f*_3_ = 4.49; γ_1_/2π = 0.223 THz, γ_2_/2π = 0.46 THz, and γ_3_/2π = 0.99 THz; *a*_1_ = 2.68×10^−2^, *a*_2_ = 1.56×10^−2^, and *a*_3_ = 1.79×10^−2^.

Our results show that the Fano profiles are fully determined by the resonance frequency, spectral lineshape, weighting factor and asymmetry factor. Figure 7 shows the normalized measurement transmission data (diamonds and circles) for our two samples with different periodicities. It is noteworthy to mention that the resonance behavior observed in the experimental results can be well reproduced with the Fano profiles (solid lines), which confirms that enhanced transmission in the resonant peak results from the coupling between surface plasmons (resonant state) and localized surface plasmons (non-resonant state)^38^.

## Conclusions

In summary, we found that super-aligned MWCNT films exhibited a strong anisotropic response in the frequency range from 0.1 to 2.5 THz. By utilizing THz-TDS, the refractive indices, permittivity, and conductivity for both *E*_**//**_ and *E*_**⊥**_ polarization were retrieved, demonstrating decreasing amplitude with increasing frequency and saturation at high frequency. A combination of the M-G and DL models was introduced to theoretically describe the experimental results, and the measurement was fitted by the M-G model in high frequencies. However, there is some small discrepancy for the perpendicular case in lower frequencies. One possible reason is the deficiency of the power of the transmitted THz pulse at low frequency, which may introduce large error in retrieving material properties. Both experimental and theoretical results reveal that our MWCNT films possess a prominent anisotropy and metallic behavior. Subsequently, periodic rectangular aperture arrays were patterned on the films with the long axis of the apertures perpendicular to the orientation of the CNTs, and showed an extraordinarily enhanced transmission with incident pulses polarized parallel to the orientation of the CNTs, while no obvious transmission was observed in the perpendicular case. The SPPs resonance frequencies at normal incidence were calculated based on the geometries of the aperture arrays exhibiting high agreement with the experiment and simulation results. Furthermore, the electric field distributions at the resonant frequencies were simulated, revealing that SPPs were excited and caused the extraordinary transmission response. Moreover, we found that the asymmetric transmission spectra line shape can be well fitted with the Fano model. These findings may enable new possibilities for realizing novel THz devices such as switches, polarizers, modulators and sensors with patterned super-aligned MWCNT films.

### Data availability

The datasets generated during and/or analysed during the current study are available from the corresponding author on reasonable request.
